# Duty to Warn: Antidepressant Black Box Suicidality Warning Is Empirically Justified

**DOI:** 10.3389/fpsyt.2020.00018

**Published:** 2020-02-13

**Authors:** Glen I. Spielmans, Tess Spence-Sing, Peter Parry

**Affiliations:** ^1^ Department of Psychology, Metropolitan State University, Saint Paul, MN, United States; ^2^ School of Clinical Medicine – Children’s Health Queensland Unit, University of Queensland, Brisbane, QLD, Australia; ^3^ College of Medicine and Public Health, Flinders University, Adelaide, SA, Australia

**Keywords:** adolescent, antidepressant, child, depression, suicide

## Abstract

The United States Food and Drug Administration issued a Black Box warning in October 2004 after placebo-controlled trials of antidepressant medications found an increased risk of suicidal thoughts and behaviors among children and adolescents taking antidepressant medications relative to placebo. Subsequently, some researchers have concluded that the Black Box warning caused severe unintended consequences; specifically, they have argued that the warning led to reduced use of antidepressants among youth, which led to more suicides. In this paper, we critically examine research regarding the Black Box warning’s alleged deleterious consequences. One study claimed that controlled trials did not actually find an increased risk of suicidality among youth taking fluoxetine relative to those taking placebo, but its measure of suicidality is likely invalid. We found that ecological time series studies claiming that decreasing antidepressant prescriptions are linked to higher rates of suicide attempts or actual suicides among youth were methodologically weak. These studies exhibited shortcomings including: selective use of time points, use of only a short-term time series, lack of performing statistical analysis, not examining level of severity/impairment among participants, inability to control confounding variables, and/or use of questionable measures of suicide attempts. Further, while some time-series studies claim that increased antidepressant prescriptions are related to fewer youth suicides, more recent data suggests that increasing antidepressant prescriptions are related to more youth suicide attempts and more completed suicides among American children and adolescents. We also note that case-control studies show increased risk of suicide attempts and suicide among youth taking antidepressants, even after controlling for some relevant confounds. As clinical trials have the greatest ability to control relevant confounds, it is important to remember such trials demonstrated increased risk of suicidality adverse events among youth taking antidepressants. The Black Box warning is firmly rooted in solid data whereas attempts to claim the warning has caused harm are based on quite weak evidence.

## History

In the modern era of evidence-based medicine, treatment decisions are presumably influenced by the available published research literature. Randomized controlled trial (RCT) data are often held up as the highest standard of evidence, as such studies can best isolate the benefits and risks of a treatment. However, observational research is also useful in examining potential adverse drug effects, though such research is unable to control for various extraneous variables. RCTs have been quite influential in swaying the opinions of clinicians, researchers, and regulatory agencies regarding the risks and benefits of antidepressant medications for youth.

Before the advent of the selective serotonin reuptake inhibitors (SSRIs), tricyclic antidepressant medications did not outperform placebo in nine small RCTs for youth depression (1). As well as this empirical lack of efficacy, tricyclic antidepressants’ lethality in overdose and adverse effects meant they were relatively rarely prescribed for children and adolescents. In contrast, during the late 1990s and early 2000s, several RCTs claimed SSRIs to be safe and efficacious in the treatment of depressed youth. Along with relative non-lethality in overdose, these RCTs provided evidence to support the quickly increasing use of SSRIs and other newer antidepressants (e.g., venlafaxine, duloxetine) for youth [e.g., (2–4)]. Interestingly, tricyclics also became more widely prescribed for youth, likely riding on the coattails of their newer counterparts (5).

However, news from RCTs was not entirely positive. In fact, many additional RCTs for youth depression had been conducted and their results were not published, likely due to finding no efficacy (6). In addition, RCT data (again, much of which was unpublished) were raising concerns among regulatory agencies regarding potential antidepressant-induced suicidality (suicidal thoughts, behaviors, and attempts). Data showing increased risk of suicidality associated with the SSRI paroxetine helped drive the Medicines and Healthcare Products Regulatory Agency in the United Kingdom to state in May 2003 that paroxetine was contraindicated in patients under age 18 (7).

Just a few days later, the United States Food and Drug Administration (FDA) provided a public health advisory against the use of paroxetine for depressed youth until additional data could be examined. In October 2003, another FDA advisory indicated that preliminary data suggested increased suicidality on antidepressants relative to placebo (7). It was clear that suicidality was coded inconsistently across trials. Thus, the FDA commissioned an external review of adverse event reports from these clinical trials. A panel of suicide experts from Columbia University examined these reports (blind to treatment assignment) using a standardized coding scheme for suicidality, the Columbia Classification Algorithm for Suicidal Assessment (C-CASA) (8). Across the 23 RCTs in youth, with access to both published and unpublished data, their results indicated a statistically significant increased likelihood of suicidality on antidepressants relative to placebo, with an odds ratio (OR) of 1.71 (95% CI: 1.14–2.77) (9). In addition, the panel found an increased risk of agitation or hostility for antidepressants over placebo (OR = 1.79, 95% CI: 1.16–2.76).

Based on these findings, the FDA issued a Black Box warning in October 2004, which was then updated in 2006 to reflect data indicating risk of antidepressant-induced suicidality in young adult patients. It states in part, that “Antidepressants increased the risk compared to placebo of suicidal thinking and behavior (suicidality) in children, adolescents, and young adults in short-term studies of major depressive disorder (MDD) and other psychiatric disorders” [(10) pg. 1]. On one hand, it is also important to note that no completed suicides were recorded in these clinical trials. On the other hand, study participants were typically receiving very regular clinical attention and, in most studies, participants with active suicidality were excluded. Thus, the finding of no completed suicides may not generalize to real-world patients. Additionally, it is important to note that suicidal ideation has been found to predict future suicide attempts among adolescents. Thus, increases in suicidal ideation are themselves concerning, even if no completed suicides occurred during the trials (11–13).

The FDA’s Black Box warning has drawn much criticism. Essentially, the warning’s detractors claim that it has resulted in fewer antidepressant prescriptions for young patients, leading to greater rates of untoward events such as suicide (14) or suicide attempts (15) due to a lack of treatment. The claim of higher rates of youth suicide subsequent to the Black Box warning seems to have originated from a 2007 study by Gibbons et al. (14). As of November 26, 2019, Google Scholar indicates this study had been cited 612 times. The Gibbons et al. study’s finding has thus taken on an air of established truth. In response to claims that the Black Box warning had negative consequences for children and youth, Sparks and Duncan critically analyzed research on youth suicide in relation to antidepressant use after the warning. Their findings negated the claims made by Gibbons et al. and other critics of the warning. They concluded that antidepressants elevate suicidality risk among youth and that the Black Box warning was empirically supported (16).

In this paper, we critically examine a sample of studies which claim deleterious consequences of the Black Box warning. In addition, we describe several methodological difficulties in linking (or unlinking) suicidality to antidepressants in ecological studies. **Table 1** provides a description of several relevant problematic claims and findings in studies which claim to have found evidence of antidepressant safety for suicide-related outcomes. Further, we discuss results from case-control studies. We also present evidence that the FDA’s Black Box warning is indeed justified and has not led to the claimed increase in youth suicidality. Several years after Sparks and Duncan’s analysis, we find that their position remains justified.

**Table 1:** Selected methodological and data reporting problems observed in the antidepressant-suicide literature.

Study	Brief description	Problematic claim/finding	Description of problematic claim/finding
Gibbons et al. (17)	Analysis of subgroup of antidepressant RCTs	Claim: antidepressants do not increase suicidality.	-Measure of suicidality (CDRS-R suicidality item) is not a sensitive, specific measure of suicidality (18–20). -Clinical raters may have lacked competence and/or relevant expertise with children/adolescents (21–24). -Number of suicidality adverse events in RCTs was higher on antidepressants than placebo to statistically significant extent (9).
Ignaszewski and Waslick (25)	Review of recent antidepressant RCTs in youth	Claim: no sign of increased suicidality on C-SSRS in three trials	-While the C-SSRS did not find excess suicidality on antidepressants, neither desvenlafaxine nor fluoxetine demonstrated efficacy. The only trial to suggest drug efficacy (26) had problems as described in the following row.
Findling (26)	RCT of escitalopram *versus* placebo	Missing analysis: no data analysis of SIQ-Jr Inaccurate data reporting: suicidality events were (inaccurately) reported for 9 of 157 participants on placebo, compared to 11 of 155 participants on escitalopram.	-Means and standard deviations are reported for the SIQ-Jr but no statistical test was performed. Our calculations indicate that placebo outperformed escitalopram on the SIQ-Jr: *t*(310) = 2.02, *p* = .045, *d* = .23. -Two of the suicidality AEs on placebo occurred *after* participants stopped taking placebo and were taking escitalopram in the community. -C-SSRS scores may also have reflected this inaccurate attribution of suicidality to placebo.
Gibbons et al. (14)	Time-series analysis of suicides in American and Dutch youth in relation to Black Box warning	Claim: youth suicides increased from 2003 to 2004 in USA, which is attributed to Black Box warning	-No statistical analysis provided for data on American youth. -Black Box warning occurred in October 2004, making it an unlikely culprit for the entire 2004 calendar year increase in American youth suicides. -Time series analyses should occur over a longer timeframe to avoid mistaking random fluctuations as meaningful. -Suicide rates in 2005 were basically unchanged from 2004, running counter to Gibbons et al.’s forecast of a continued substantial increase in USA youth suicides. -There is no attempt to control for any extraneous variables which may affect suicide rates.
		Claim: inverse correlation between antidepressant prescriptions and suicide rate in Dutch youth between 1998 and 2005	-Small numbers of suicides among Dutch youth are subject to much yearly variation. -Subsequent data 2006 shows a decrease in Dutch youth suicides along with an increase in antidepressant prescriptions, contrary to Gibbons et al.’s forecast. -This particular time window (1998–2005) may not be representative of a wider time series. -There is no attempt to control for any extraneous variables which may affect suicide rates.
Lu et al. (15)	Ecological study examining relationship between antidepressant prescriptions and suicide attempts in American youth	Claim: decreased antidepressant prescriptions were associated with more suicide attempts among youth.	-Antidepressant prescriptions changed by less than one percentage point during the study -Their proxy measure of “suicide attempts” was “poisoning by psychotropic agents.” The citation they provide for the validity of this proxy measure is Patrick et al. (27). Two of the authors of Partick et al. (28) wrote a response to Lu et al. (15) noting that a) this proxy measure is not a good measure of suicide attempts b) five other studies found no evidence of increased suicide attempts or suicides among youth in relation to a flattening rate of antidepressant prescriptions -It seems illogical that decreased rates of antidepressant prescriptions would cause increased risk of poisoning *via* psychotropic drugs.
Isaacson and Ahlner (29)	Ecological study examining relationship between antidepressant prescriptions and suicide in Sweden	Increase in suicide after 2003 was “remarkable”	-Increase in suicides is based on small absolute numbers and a small increase from 2003 to 2007. -Depending on the years included in an analysis, one could draw a variety of conclusions regarding antidepressants and suicide from the same dataset.
		Few youth who committed suicide had a recent antidepressant prescription (20%) or tested positive on a toxicology exam (12%)	-This observation does not rule out antidepressant withdrawal as a precipitant for suicide. -Another study found that 39% of Swedish women (ages 15–24) who committed suicide from 2009 to 2013 tested positive for an antidepressant on a toxicology exam (30).
Katz (31)	Ecological study examining relationship between antidepressant prescriptions and suicide rate in Manitoba, Canada	The rate of completed suicides increased to a statistically significant extent after regulatory warnings	-Aboriginal youth comprised a disproportionately high percentage of completed suicides—and it seems unlikely that many Aboriginal youth were taking antidepressants before the warning. -The decreased use of antidepressants likely occurred mainly among non-Aboriginal youth.

### Clinical Trials

#### Re-Analysis of Clinical Trial Data

Based upon re-analyzing data from selected RCTs, Gibbons and colleagues concluded that antidepressants both possessed substantially more efficacy than had been seen in prior analyses (32) and posed no risk of increasing suicidality in youth (17). Given our paper’s focus on the Black Box warning, readers are directed elsewhere for critical examination of the Gibbons et al. paper focused on purportedly greater antidepressant efficacy (33–35). One obvious shortcoming of their analysis is that only data from fluoxetine trials were included. This is problematic given that a large majority of antidepressant trials in youth have used drugs other than fluoxetine. Further, at least one of the included fluoxetine trials was only “approximately 3 weeks in length” despite Gibbons et al.’s claim that all participants were treated with fluoxetine for at least 6 weeks [(35) pg. 1].

The Gibbons et al. suicidality paper used an item on the Childhood Depression Rating Scale-Revised (CDRS-R) as a measure of “suicide risk.” Their exact definition of treatment-emergent suicidal ideation was a CDRS-R score of greater than two, which would indicate thoughts about suicide, typically when angry, or anything more severe (such as suicidal threats or attempts). The FDA disapproves of using a depression rating scale item to assess suicide risk. Indeed, the longtime director (now retired) of the FDA’s Psychiatry Products Division, Thomas Laughren stated that such rating scales “turned out not to be very helpful” in detecting suicidality [(18) pg. 343]. Similarly, Robert Temple, another senior official at FDA has noted that a depression rating scale is “not where it shows up,” in reference to suicidality among youth taking antidepressants (19). Lending empirical support to the concerns of Laughren and Temple, GlaxoSmithKline performed an analysis of suicidality-related events in its pediatric trials of paroxetine. Similar to the aforementioned FDA analysis, reviewers who examined adverse events reports were blinded to treatment assignment. They found that suicide-related events occurred in 3.4% of paroxetine-treated patients relative to 0.9% of patients taking placebo, a statistically significant (and clinically concerning) difference (20). Yet on the depression rating scale items pertaining to suicidality, there was no difference between paroxetine and placebo, suggesting these items are unable to detect increased rates of suicidal events (20).

Another problem with relying on rating scale items is the lack of training/competence of clinical trial raters. In a rather scathing review, John Walkup pointed to several problems in industry-funded investigations of antidepressants for children and adolescents. Most germane to the current paper, he states that these RCTs were hastily arranged, using clinical raters who were not sufficiently trained in administering and properly scoring rating scales and who may have lacked experience with children and adolescents (36). We are unfamiliar with any studies directly assessing rater competence in these trials, but a small body of literature suggests that raters in adult psychiatry clinical trials are often not particularly adept at administering rating scales (21–23). For instance, one study examined recordings of Hamilton Depression Rating Scale (HAM-D) administrations across two clinical trials of antidepressants in adults (22). In 81% of HAM-D interviews, there was inadequate use of follow-up questions to obtain sufficient information and 64% of HAM-D administrations did not adequately clarify ambiguous participant responses. There is a small body of literature expressing concerns regarding suboptimal application of clinical ratings in trials (21–24, 36); we are unaware of any empirical investigations finding that such ratings are typically done to a high standard of competence. We see no reason that these problems would not also extend to pediatric trials; indeed, having raters who are not well-versed in working with youth would likely detract from the reliability/validity of CDRS-R or other rating scale scores.

Gibbons et al. claim an absence of drug-induced suicidality using a measure that does not clearly detect suicidality (20) administered by raters of questionable competence (21–23, 36). This fails to override results based on actual reports of suicidal adverse events adjudicated *via* blinded raters using the C-CASA coding system (9). Thus, the re-analysis of a small sample of fluoxetine clinical trials by Gibbons et al. (17) does not advance understanding of antidepressant-related suicidality in youth.

#### Treatment of Adolescents With Depression

While Walkup’s aforementioned review takes aim at industry-funded antidepressant RCTs for youth, he states that the National Institute of Mental Health (NIMH)-funded trials on treating adolescent depression utilized much stronger methodology. These studies were publicly funded and were run by researchers with “bona fide expertise” in both depression and clinical trial methodology [(36) pg. 3]. Investigators, clinicians, and evaluators were described as having frequent discussions to maintain “fidelity and quality” [(36) pg. 4]. The largest NIMH-funded trial, the Treatment of Adolescents with Depression (TADS) study is indeed worth strong consideration when examining the risk-benefit ratio of relevant treatments. In TADS, a total of 439 depressed adolescents were randomly assigned to one of four arms: fluoxetine (n = 109), placebo (n = 112), cognitive-behavioral therapy (CBT, n = 111), or combined CBT + fluoxetine (n = 107). The first 12 weeks constituted the RCT phase of the study. After the first 12 weeks, treatment assignment was unblinded and a 24-week open-label follow-up phase ensued. In this follow-up period, several participants who initially took placebo were switched to CBT or fluoxetine. Adverse events related to suicidality were recorded and analyzed using the C-CASA to maximize accurate classification of such events.

However, despite use of the C-CASA, descriptions of the number of TADS suicidal events are challenging to parse, as they vary both across and within publications of safety data from the trial (37, 38). For instance, in the main safety publication from the RCT phase of the TADS study, suicidality events were reported as occurring among 10 (9.2%) participants taking fluoxetine relative to three (2.7%) participants taking placebo, five participants (4.5%) receiving CBT alone, and five participants receiving combined CBT + fluoxetine (4.7%). The difference between fluoxetine and placebo was noted as statistically significant (39). A subsequent publication from the TADS team was titled “Suicidal Events in the Treatment for Adolescents with Depression Study,” and appears to be the main TADS team publication focused on suicidality (40). In the paper, the authors do not mention that the risk of suicidal events was significantly higher on fluoxetine than placebo during the RCT phase of the trial—no statistical comparison of such events between drug and placebo is provided (40). Given the topic of the paper, this omission is remarkable. Rather, the suicidality paper reports on total suicidal events across the course of the 36 weeks of the study, combining the RCT and open-label treatment phases. By collapsing the data from the open-label phase with the RCT phase, the composition of the TADS placebo group was compromised and the statistically significant increase in suicidality on fluoxetine was lost.

The authors reported that 12 placebo participants (10.7%) had an incident of suicidality, whereas the number was slightly, but not significantly higher among participants who were initially assigned to take fluoxetine (14.7% of participants, n = 16) (40). Participants taking placebo went from having three suicidal events in one publication to 12 suicidal events in another. These differing numbers are explained thusly: “Some patients who had been randomized to … PBO (N = 9) and had a suicidal event *were in fact on SSRI medication at the time* (italics added) for (sic) the event having started antidepressant treatment because of non-response to the randomly assigned treatment [(40) pg. 744].” The number of 12 comes from all participants who were initially assigned to take placebo. Three participants experienced reported suicidal events while taking placebo, while four placebo participants dropped from the RCT and had a suicidal event while taking medication in the community during the initial 12 weeks of the study. Five additional participants randomized to placebo experienced a suicidal event *while taking medication after the placebo-controlled phase was over*. This odd data reporting maneuver drastically inflates the apparent risk of placebo, thus deflating the apparent suicidality risk incurred by medication. Underneath their **Table 1**, and not described in the body of the paper, the authors note that when the analysis is limited to events occurring when actually taking study-prescribed CBT, placebo, or fluoxetine, the risk for suicidal events is higher for fluoxetine compared to either CBT (p = .04) or placebo (p = .02) (40).

Further complicating matters, seven participants had more than one suicidal event—but only their first suicidal event is reported. Thus, the TADS paper on suicidality—which one might expect to provide information on all suicidal events—underreports and obscures the total number of suicidal events (38–40). The suicidality paper performed some useful analyses, examining such matters as whether drug–induced behavioral activation is related to suicidality and whether risk for suicidality changes over the course of treatment. While such analyses are welcome, a clearer and more thorough reporting of actual suicidal events would also be vitally important.

If TADS is indeed the most reliable study on treatment for depressed youth, then it should be concerning that fluoxetine led to substantially more suicidal events relative to CBT or placebo. Despite the limited statistical power to detect an uncommon adverse event, the increase in suicidal events on fluoxetine was statistically significant. Further, it is troubling that this important finding was obscured in a publication from one of the most qualified and competent teams to study the risk of suicidality in an antidepressant RCT for youth.

#### Recent Clinical Trials

Since the FDA issued its Black Box warning, a handful of additional trials have been conducted. In some of these trials, the Columbia Suicide Severity Rating Scale (C-SSRS) was used to detect suicidality. Rather than just representing a small section of a depression rating scale, the C-SSRS focuses solely on suicidality and asks several specific questions regarding ideation, suicidal planning, and suicidal behavior (8). Importantly, the C-SSRS attempts to assess suicidal planning and intent, two important areas that are key to a detailed understanding of suicidality (41). This measure seems to be more thoroughly validated in detecting contemporary suicidal events and predicting future suicidal events than suicidality items on depression rating scales described earlier (42, 43). While the C-SSRS is undoubtedly an improvement over prior unsystematic methods of assessing suicidality, there remains some disagreement about its overall utility (44, 45).

A recent paper by Ignaszewski and Waslick claims that three newer acute-phase trials using the C-SSRS show no sign of antidepressant-induced suicidality and “may suggest that treatment-emergent suicidality is not as significant a risk as indicated” by the FDA [(25) pg. 2]. Our review of the three trials (two focused on duloxetine, one on escitalopram) confirms that the C-SSRS shows very similar percentages of treatment-emergent suicidal ideation and behavior between drug and placebo (26, 46–48). Suicidality-related adverse events were recorded at slightly less than 1% of drug and placebo participants in the two duloxetine trials, which also included a fluoxetine arm (47, 48). Neither duloxetine trial found that duloxetine demonstrated efficacy over placebo. Escitalopram showed statistically significant superiority over placebo on the CDRS-R (26, 46). We have concerns with some aspects of the methodology and data reporting in these papers.

In the escitalopram trial, suicidality-related adverse events were reported in 11 of 155 participants on escitalopram compared to 9 of 157 participants on placebo. However, papers reporting on both the acute-phase and placebo-controlled extension phase misattributed a suicidality-related adverse event to a separate placebo participant in each phase (26, 46). In both cases, the participant assigned to placebo dropped out of the study, then had a suicidal event while taking escitalopram in the community. Given that these participants had dropped from the study and were taking medication (not placebo) at the time of the event, these events should not be attributed to placebo. If one correctly subtracts these 2 suicidality events from the 9 events attributed to placebo, there were 7 events on placebo relative to 11 events on medication. The C-SSRS was administered at all study visits. If the study was able to gather data on adverse events for these two placebo participants who discontinued placebo, study protocol would also dictate C-SSRS administration. If the authors misattributed suicidality adverse events to placebo, it seems likely that these suicidal events were also misattributed to placebo on the C-SSRS ratings. If corrected, this would lead to 12/128 placebo participants (9.4%) having suicidality events on the C-SSRS compared to 19/131 (14.5%) on escitalopram. Granted, this represents some speculation on our part. Such speculation would be unnecessary if adverse events were not clearly misattributed to placebo.

Typically, antidepressant trials have excluded participants who present with concerns regarding suicidality. Some studies have allowed participants without “significant” suicide risk to participate. Across the two newer duloxetine studies (which both included a fluoxetine comparison group), participants who exhibited suicidality and took placebo had a non-significantly greater likelihood of experiencing reduction in suicidal ideation during the course of the trial (94.12% of placebo participants relative to 81.61% of participants taking medication (47, 48). Obviously, this is tentative and in need of more study, but does not exactly suggest anti-suicidal properties of antidepressants. In the escitalopram trial, participants completed the Suicidal Ideation Questionnaire—Junior High School Version [SIQ-Jr, a questionnaire examining suicidal thoughts and intentions (49)]. Participants showed more improvement on the SIQ-Jr on placebo than on escitalopram (26). While the relevant paper reports the means and standard deviations for this measure, it did not conduct a statistical comparison between groups (26). The authors do not provide the sample size for this measure. For the SIQ-Jr, we used the same sample size as was provided for the primary outcome measure, as the study methods describe using an intention-to-treat approach to analysis, so data from all participants would be captured for each measure. Based on this information, we conducted a t-test and found that placebo outperformed escitalopram to a statistically significant and small degree [t (310) = 2.02, p = .045, d = .23]. The lack of reporting a statistical comparison between drug and placebo is unjustifiable.

In addition to manufacturing escitalopram, Forest also sponsored the antidepressant citalopram. Data reporting in a RCT of citalopram in depressed youth is relevant to our above observation that the SIQ-Jr was not statistically analyzed in Findling et al.’s escitalopram trial, which was sponsored by Forest (26). One trial of citalopram was known as CIT-MD-18. During the trial, nine participants took citalopram that was mistakenly administered in an unblinded manner. The study protocol stated that if the blind were broken for any particular patient, that patient would be excluded from any efficacy analyses. Yet the main journal article incorrectly included eight of these unblinded participants in the statistical analysis of the primary outcome measure, on which citalopram outperformed placebo to a statistically significant degree (p = .038) (50). However, Jureidini et al.’s review of internal Forest documents reveals that if protocol were followed and these eight participants would have been excluded, then the p-value on the primary outcome marginally missed the mark of statistical significance (p = .052) and citalopram was no longer statistically superior in efficacy to placebo (51). Further, the journal article a) reported positive results on some secondary outcomes that were not listed in the statistical analysis protocol and b) failed to report the negative results on some secondary outcomes which were listed in the statistical analysis protocol (51). Thus, our observation of incomplete data analysis on the SIQ-Jr in Findling et al.’s escitalopram trial (26) aligns with problematic data reporting in another paper describing clinical trial outcomes for another antidepressant sponsored by Forest (51).

TADS also used the SIQ-Jr to assess suicidality. In TADS, SIQ-Jr baseline scores were predictive of treatment-emergent suicidal events whereas baseline CDRS-S total scores and CDRS-R suicidality items were not. The TADS suicidality paper stated that “youths appears more likely to indicate clinically relevant suicidal ideation when completing an assessment questionnaire themselves rather than to share these thoughts in a direct interview with a clinician” [(40) pg. 746], lending credence to our concern with the self-reported SIQ-Jr showing results unfavorable to escitalopram.

We also examined two recent desvenlafaxine trials for youth depression, neither of which demonstrated efficacy for desvenlafaxine (52, 53). Across the two trials, we did not observe clear trends in suicidality as reported *via* adverse events or on the C-SSRS.

The data in all of these recent trials do not provide a consistent signal of suicidality, but placebo showing more improvement than escitalopram on SIQ-Jr suicidal ideation scores raises concern. It is also problematic that suicidality events were misattributed to placebo (26, 46). We do not see these newer trials as vindicating antidepressants in terms of suicidality risk.

### Selective Publication of Data

In addition to the above concerns, it has been demonstrated that data reported in clinical trials is sometimes incomplete or inaccurate—and not only in the citalopram and escitalopram studies described above. An illuminating review by Hughes et al. compared data on reported suicides and deaths in clinical trials between two sources: online clinical trial registries maintained by drug firms and journal articles that reported outcomes from the same trials. Across 142 trials of six psychiatric medications, 62.3% of deaths, 53.3% of suicides, and 46% of suicidal ideation/attempts/injury events that appeared in the online registries did not appear in the published journal articles (54). Sharma et al. examined clinical study reports of duloxetine, paroxetine, sertraline, and venlafaxine (55). They found that adverse events were sometimes labeled as “worsening depression” or “emotional lability” when in fact the relevant descriptive narratives in the clinical study reports clearly describe suicide attempts.

One might argue that the FDA process of adjudicating suicide-related events in antidepressant clinical trials in youth resolves problems of publication bias or mislabeled adverse events. We are not so certain. One trial, known as Study 329, sponsored by GlaxoSmithKline, compared paroxetine (n = 93), imipramine (n = 95), and placebo (n = 87) for depressed adolescents. In the original Keller et al. article published in the *Journal of the American Academy of Child and Adolescent Psychiatry*, a total of five suicidality events were recorded for paroxetine, three for imipramine, and one for placebo—and these events were mainly described under the euphemistic term “emotional lability” (2). In the FDA’s subsequent analysis of antidepressant-related suicidality, eight events on paroxetine were coded as indicative of suicidality in Study 329 (9).

Several years after the FDA’s analysis of the paroxetine study, Le Noury and colleagues conducted an independent re-analysis of the original trial data. Le Noury et al. underwent a lengthy process to get paroxetine’s sponsor (GlaxoSmithKline) to provide access to raw data on case report forms *via* an ungainly data portal which did not allow the researchers to print or download the data (56). Le Noury et al. then examined the clinical study report and appendices, along with these individual case report forms. During the acute phase of the trial, they coded 10 suicidality events as occurring on paroxetine, three definite and one possible suicidality events on imipramine, and one definite and one possible suicidality event on placebo (57). These researchers provide a rationale for their coding of these cases in an appendix (57). We find their coding of suicidality in these two additional cases (beyond the Columbia/FDA coding) to be reasonable. It is quite interesting to note that Le Noury et al. found double the number of suicidality events on paroxetine compared to those reported in the original journal article reporting trial results (2). Their coding also yielded two more suicidality events on paroxetine than were found in the FDA-sponsored analysis.

We are unaware of whether any other suicidality events were miscoded (e.g., worsening depression, intercurrent illness, or other such terms) in antidepressant trials other than Study 329 and thus possibly escaped detection in the FDA suicidality analysis. Appendices in the FDA suicidality report describe how drug firms were asked to provide data on cases of possible suicidality (9). The FDA request seems thorough in casting a wide net for most forms of suicidal behavior though it was perhaps less thorough in assessing possible ideation (9). While the FDA requested data, it was the duty of the drug firms to report their data accurately. Given the industry’s spotty track record in reporting adverse events, one cannot exclude the possibility that all suicidality events may not have been reported. Certainly, using the C-CASA algorithm to sort through reported adverse events improved the reliability of suicidal event coding. Further, use of such ratings as the C-SSRS and SIQ-Jr are welcome additions to the clinical trial literature. However, there likely remains room for improvement in reliably assessing suicidality during clinical trials.

### Ecological Studies

Many studies have examined correlations between antidepressant prescriptions and suicide rates at a national population level. A 2007 review described eight of 19 such studies finding that suicide rates decreased as antidepressant prescriptions increased and no studies finding that suicides increased along with increased antidepressant prescribing (58). However, national data on suicide rates are not necessarily accurate. Autopsies are not always performed, and in many deaths it is impossible to detect suicidal intent with certainty (e.g., drug overdose, fatal automobile crash, etc.). Whether a death is coded as suicide varies across countries; relatedly, lower autopsy rates are related to lower rates of deaths coded as suicide (59). That being said, autopsy rates are likely higher among youth, so perhaps more faith can be placed in the accuracy of population-level suicide data among children and adolescents (60). Risk for youth suicide is heavily multifactorial (61). Many relevant variables (parental unemployment, alcohol/drug use, family dysfunction, access to firearms, parental military deployment, etc.) are typically not controlled for in studies attempting to link population-level antidepressant use to suicide rate. Additionally, such studies are unable to examine whether antidepressant withdrawal may precipitate suicide (62, 63). Despite our concerns with the methodology of ecological studies, we describe a handful of them here, as such research is often cited in the debate about the benefits or harms of antidepressant suicidality warnings.

#### Selective Time Points Distort Data

The claim of higher rates of youth suicide subsequent to the Black Box warning appears to have originated from the 2007 study by Gibbons and colleagues (14). One might expect that such an influential paper would consist of well-reasoned conclusions based on solid research methodology. However, Gibbons et al. did not actually statistically analyze data on suicidality in relation to antidepressant usage among American youth in this study. Rather, the authors simply report on the relationship between antidepressant prescriptions and suicides in American youth over a short time span. They observe that “In the United States, youth suicide rates increased by 14% between 2003 and 2004, which is the largest year-to-year change in suicide rates in this population since the Centers for Disease Control and Prevention began systematically collecting suicide data in 1979” (pg. 1356). Though an increase in suicide occurred after the FDA advisories in 2003, the actual Black Box warning did not occur until October 2004. It thus seems difficult to blame the October 2004 Black Box warning for an increase in suicides for the entire year of 2004. Gibbons et al. also state: “Given that SSRI prescriptions for children under age 15 already underwent a reduction of approximately 17% from 2003 to 2005, we expect an increase of 0.11 suicides per 100,000 children in this age group. Since there are approximately 40 million children in this age group, we would expect 44 additional deaths by suicide in 2005 relative to 2003, or an increase of 18% in this age group” (pg. 1361). The predictions of the authors were wrong; a letter to the editor noted that suicide figures for 2005 did not show a substantial increase from 2003 (64). But even if their prediction would have been exactly correct, the study would nonetheless have been highly flawed. Any serious time-series analysis should analyze data over several time points, so that random fluctuations in data are not mistaken as meaningful trends. Other variables that influence suicide rates should also be controlled—a challenging task outside of a clinical trial.

In the same paper, the authors also examined data from the Netherlands. They note that there was a 22% decline in child and adolescent antidepressant prescription rates along with a 49% increase in suicides over the period of 2003–2005. Between 1998 and 2005, the inverse relationship between antidepressant prescriptions and suicide rate among Dutch youth was described as “significant.” In an article in the Dutch Drug Bulletin [as reported in (65), pg. 112], Dutch researchers noted the increase in raw suicide numbers was from 34 in 2003 to 51 in 2005. The editor of the Dutch Drug Bulletin stated: “The (suicide) numbers for the Netherlands are so small that you have to be very, very careful before you make a statement” (pg. 112) adding that he thought Gibbons et al.’s claims were “reckless” (pg. 112). Further, 2006 data on suicide among Dutch youth found a slight drop to 48 suicides. Data from a study published several years afterward found that this slight drop in suicides occurred along with a decrease from 2005 to 2006 in terms of youth antidepressant prescriptions in the Netherlands (66), not the result which Gibbons et al. would have forecasted. Though often cited as evidence of regulatory warnings leading to increasing suicides, Gibbons et al. provided weak evidence. As will be demonstrated later in this paper, more careful data analysis over longer time periods reaches different conclusions.

#### Mismeasuring Suicide Attempts

Another investigation purportedly linked decreasing antidepressant prescriptions to increased rates of suicide attempts in the United States (15). But the study did not actually measure suicide attempts. Rather, they used “poisoning by psychotropic agents” as their proxy measure of suicide attempts. Further, they emphasize “relative changes” in antidepressant use—which distracts from their finding that antidepressant use among youth in their sample basically flatlined (rather than plummeted) during the FDA warning period. The change in prescription rate was *less than a percentage point*. Rates of completed suicide were unchanged. There is also a seeming logical problem in their analysis that claims a lack of antidepressant use causes poisoning *via* psychotropic agents. If fewer patients are receiving treatment due to the regulatory warnings, then they would likely have a more difficult time obtaining psychotropic drugs on which to overdose.

Lu et al. cite one study as validating their choice of “poisoning by psychotropic agents” as a proxy for suicide attempts (27). Two authors of this validation study (Barber and Miller) and an additional co-author (Azrael) wrote a response to Lu et al.’s study (28). Barber et al. state that Lu et al.’s findings regarding suicide attempts are likely based on their “unusual proxy” measure which Barber et al. describe as actually having insufficient sensitivity (40%) for detecting actual suicide attempts. They also state that a third of psychotropic poisonings were not actually suicide attempts in the validation study cited by Lu et al. A weak measure of suicide attempts, indeed. Further, Lu et al.’s description of the validation study was quite selective. The validation study found that psychotropic poisoning fared better as a proxy measure for suicide attempts in British Columbia, Canada than in the United States, with positive predictive values of 79.7 and 67.2%, respectively. In justifying their selection of psychotropic poisoning as a stand-in for suicide attempts among American patients, Lu et al. describe data from the Canadian portion of Barber et al.’s study without describing the less favorable results from the American patients in the same study. To sum, a) citing more favorable validity statistics based on Canadian patients, while b) ignoring less favorable validity data from American patients to c) shore up one’s selection of an odd proxy measure for suicide attempts suicide in a study of American patients is illogical and of questionable methodological rigor.

Barber et al.’s response also noted five other data sources showing no increase in either suicide attempts or completed suicides contemporaneously with the flattening prescription rate of antidepressant drugs (28). Lu et al.’s paper generated headlines such as “Warnings on headlines may have backfired” (67) and “Warnings against antidepressants for teens may have backfired” (68). However, these headlines are not justified by the Lu et al. study.

#### Other Ecological Studies

Numerous ecological studies outside of the United States have investigated the contested link between antidepressant use and suicide. Dahlberg and Lundin conducted one of the more rigorous ecological studies to examine the relationship between antidepressant use and suicide (69). They examined the rate of suicides and antidepressant sales from 1990 to 2000 using suicide data from the Swedish National Health Board, and data on antidepressant sales from Apoteket AB (the Swedish government prescription drug retailer). Variables in the analysis included antidepressant sales, suicide, gender, age group, county, year, unemployment, and alcohol sales. When including only two variables in their analysis (suicide and antidepressant sales) for all ages, they found that increased antidepressant sales predicted less suicide. When they controlled for additional variables including socioeconomic characteristics (rates of unemployment and alcohol sales), the correlation was no longer significant.

When they analyzed the data by age group, the researchers found that higher antidepressant sales were associated with a higher rate of suicide for youth (up to age 25). They also found that across all ages, the increase in antidepressant sales was larger for women, while the decrease in suicides was larger for men. Overall, they did not find that suicides significantly decreased with increasing antidepressant sales, and they found a significant association between increasing antidepressant sales and increasing suicide for youth (up to age 25 years).

It is important to re-emphasize that when only the two main variables—antidepressant use and suicide—were analyzed, the results seemed to show that increases in antidepressant use were associated with decreases in suicide. However, this finding did not hold when other key variables were controlled for. These findings highlight the multifactorial nature of youth suicide risk and provide a cautionary reminder to remain skeptical regarding the findings of correlational studies that fail to control for other important risk factors in youth suicide. Going a step further, while statistical controls for confounding variables increase the validity of ecological studies, it would be impossible to control for all relevant confounds in such research.

Another ecological study investigated whether warnings on antidepressants for youth were related to changes in youth suicide in Sweden. Isacsson and Ahlner (29) compared suicides and antidepressant use in Sweden from two periods for youth aged 10 to 19 years; period 1 was from 1992 to 2002, and period 2 was from 2003 to 2010 (29). They state that youth suicides increased for 5 years after the Black Box warning. Based on evidence from toxicology reports, they argue that the majority of suicides occurred without use of antidepressants.

This paper reports data in an idiosyncratic manner. Period 1 is from 1992 to 2002, but it appears that they only have information on antidepressant use starting in 1999. That is, in period 1, they only have data on both antidepressant use and suicides for 4 years—from 1999 to 2002. They call the increase in suicide after 2003 “remarkable” (pg. 299), although it was a slow incline based on a very small number of completed suicides from 2003 to 2007, with suicides increasing from 43 in 2003 to 59 in 2007. The number of suicides then increases alongside an increase in antidepressant prescriptions from 2006 to 2009, though these relevant data are not noted in the text of their paper. Further, there are dramatic percentage changes in suicides across some years; i.e., in 1998–1999 (from 29 to 46 suicides) and in 2001–2002 (from 30 to 47 suicides). There is no explanation provided for these yearly increases in suicides, which do not occur in tandem with decreasing antidepressant use. If one were to use Gibbons’ methodology of making conclusions based on 1-year snapshots, one could conclude that antidepressants have a devastatingly high impact on *increasing* risk for suicide (14). Indeed, depending on which particular groups of years were selected, nearly any conclusion regarding antidepressants and suicide could be drawn. Of course, as noted above, we do not approve of such methods. We do find it interesting that such selective and unjustifiable data interpretations have been cited many times to claim the *safety* of antidepressants—one can easily cherry-pick such data *via* selection of start and end dates in a time series analysis to support any conclusions that one wishes.

Isacsson and Ahlner found that only 20.1% of youth who committed suicide from 2006 to 2010 had received an antidepressant prescription during their last 6 months alive, with a total of 12.2% of those who committed suicide testing positive on their toxicology examination (29). This does not provide information regarding whether drug discontinuation effects may have been a risk factor for suicide (62, 63). Further, in contrast to Isacsson and Ahlner’s findings, a subsequent study regarding toxicology reports of 15–24 year old Swedish women who committed suicide found that 39% of cases in 2009–2013 tested positive for an antidepressant during a post-suicide forensic examination (30).

Wheeler et al. sought to investigate whether the rates of suicide and serious self-harm in youth changed after the United Kingdom’s regulatory warnings regarding SSRIs were issued in 2003 (70). To do so, they examined rates of SSRI prescribing, suicide, and hospitalizations due to self-harm in youth from 1993 to 2006. The researchers looked for temporal associations between trends in the rates of antidepressant prescriptions and the rates of suicide and hospitalizations due to self-harm.

They obtained data on SSRI prescribing from IMS Health for the years 1993 to 2006, and data on suicides from the Office for National Statistics for youth 12 to 17 years old from 1993 to 2005. The Department of Health provided data regarding hospitalization due to self-harm or ‘events of undetermined intent’ for youth 12 to 17 years old from 1999 to 2006. Prescribing data were for the UK, suicide data was for England and Wales, and hospitalization data was for England only.

Wheeler et al. found that after 2003, there was a steep decline in antidepressant prescribing. Sixty-five percent of the overall decline in antidepressant prescriptions was for SSRI prescriptions. The decrease in antidepressant prescription rate between 2003 and 2005 was between 40 and 50%. The decline in antidepressant prescriptions was substantially larger among British youth than for American youth during the post-warning period. Thus, if decreased population-level antidepressant prescription causes more youth suicides, one would expect to see a substantial increase in UK suicide rates during this time period. While the rate of antidepressant prescriptions declined, the trend for the overall suicide rate in youth, which had been on a consistent decline from 1993 to 2005, was unchanged. Statistical analyses confirmed that there were no shifts in the trend in the suicide rate after 2003. Wheeler also analyzed suicide data by sex, and neither the male nor the female suicide rate appeared to have been affected by the warnings. The suicide rate for young males began to decline in 2001. The suicide rate for female youth was also declining overall, though the trend was characterized by greater fluctuations due to fewer suicides for females. Wheeler at al. found a continuous, stable increase in the rate of hospitalization due to self-harm for females starting in 1993; there were no changes in the trend after the warnings. For males, the rate of hospitalization was stable. Thus, Wheeler et al. did not find evidence to support a temporal link between the regulatory warnings and suicidality in the U.K.

Wheeler et al.’s findings show, once again, the difficulty in linking trends in youth suicide rates to the rate of antidepressant use. Numerous factors, which are usually unmeasured and uncontrolled for in ecological and observational studies contribute to fluctuating suicide rates. While we are cautious when interpreting the findings of a study that did not measure or account for other factors that may contribute to the suicide rate, the study did not find evidence to support claims that declines in antidepressant use precipitate increases in suicide. Wheeler et al.’s findings have methodological advantages over the contemporaneous findings of Gibbons’ et al., particularly with a much longer period of data collection and a much steeper decline of antidepressant prescribing to youth following the 2003 warnings, yet Google Scholar indicates just 86 citations by November 26, 2019 compared with Gibbons et al.’s 612 citations.

Katz et al. examined the relationship between antidepressant prescription rate and suicides among youth in Manitoba, Canada (31). They found that physician visits due to depression in youth decreased after the relevant antidepressant regulatory warning in Canada in 2004. Between 1995 and 2005, there were only 99 completed suicides among children and adolescents, so the total sample of relevant deaths was quite small during any individual year. While the rate of suicide attempts did not change post-warning, the number of completed suicides among youth increased to a statistically significant extent.

Briefly reported in the text of the article, but not discussed, was that Aboriginal youth accounted for 74 to 85% of completed suicides between 2003 and 2005, and that the percentage of suicides occurring on an Aboriginal reserve was 73% in 2003 and 57% in 2005. Indeed, suicide rates are so alarmingly high among Aboriginal youth in Canada that the Canadian government recently created the National Aboriginal Youth Suicide Prevention Strategy to systematically address this tragic problem. Among Manitoban youth who committed suicide from 1988 to 1994, 21.9% of non-Aboriginal people had received psychiatric care beforehand compared to only 6.6% of Aboriginal people (71). We thus find it unlikely that many Aboriginal youth were receiving antidepressant treatment prior to the warning. Indeed, it seems quite possible that Katz et al. simply found that the two following events occurred alongside each other: a) an increase in suicides, mainly among Aboriginal youth and b) a reduction in antidepressant treatment, mainly among non-Aboriginal youth. A longer time-series and studying a larger population (with a larger number of suicides) would strengthen the ability to draw conclusions. Further, the extent to which Western biomedical conceptualizations and treatments are appropriate for understanding and treating Aboriginal mental health problems has been thoughtfully called into question (72, 73). Indeed, it is questionable whether antidepressants are a viable solution to the high suicide rate among Aboriginal youth. In the face of longstanding structural racism and oppression, dispensing antidepressants rather than implementing both culturally-sensitive approaches to mental health and broader systemic change may, even if occasionally efficacious, simply serve to individually pathologize Aboriginal youth.

#### Longer-Term Trends After Regulatory Warnings

When acknowledging that his prediction about a substantial increase in American teen suicides post-Black Box warning was incorrect Gibbons stated that “…the real question is whether the most severely ill children who are at greatest risk of suicide are receiving treatment for their illness. It is possible that changes in the overall population-level antidepressant treatment rate may not accurately reflect the rate of antidepressant treatment among those children at greatest risk for suicide” [(74) pg. 1910]. This insightful comment serves as a reminder that population-level data provide little insight into which particular individuals are receiving treatment. Fortunately, Gibbons’ concern has been addressed. Kafali et al. examined data from the Medical Expenditures Panel Survey (MEPS) from 2000 to 2011 on children ages 5–17 (75). MEPS is considered a representative sample of the non-institutionalized US population. The researchers divided the 12-year timespan into four segments: early prewarning (2000–2001), prewarning (2002–2003), early postwarning (2004–2007), and late postwarning (2008–2011). Parents of the participating children completed the Columbia Impairment Scale (CIS), a brief global measure of their child(ren)’s level of impairment. Data on antidepressant prescriptions were available for all participants.

While antidepressant prescription rates for youth dipped to a statistically significant extent (from about 2.25% of youth to about 1.75%) in the early postwarning period, they returned to prewarning levels by 2009. Among children whose parents had indicated they had severe psychological impairment on the CIS, there was no decrease in antidepressant prescriptions after the FDA warning. The temporary drop in antidepressant prescriptions among youth was driven solely by reduced prescriptions for youth whose parents did not rate as having severe impairment. Thus, the concern that the Black Box warning led to the most vulnerable patients being undertreated with antidepressants is simply not borne out by empirical inquiry. Indeed, it seems that clinicians responded to the warning in a reasonable fashion. In viewing antidepressants as increasingly risky due to the warning, they prescribed them less often to the people who had the least need for them while still maintaining the same level of treatment with the most impaired depressed youth, albeit with likely quite limited efficacy based on RCT findings (76, 77). The subsequent return to prewarning rates of antidepressant use in the later postwarning years suggests that the Black Box warning’s impact did not last, contrary to claims made by several researchers who have suggested that the warning has led to decreased antidepressant prescription rates.

Valluri et al. investigated the effects of the March 2004 FDA suicidality warning on antidepressant use in the treatment of youth with new-onset depression (78). The researchers analyzed a) the relationship between the warning and the use of antidepressants and psychotherapy in the treatment of children and adolescents, and b) whether the impact of the warning differed for children with major depressive disorder and those with less severe depression diagnoses. They analyzed data on antidepressant use from July 2003 to December 2006 for 40,309 American children and adolescents with new-onset depression in a repeated measures, longitudinal design. While Kafali et al. studied long-run trends in antidepressant use after the Black Box Warning (2004–2011), Valluri et al. observed changes in treatment only in the early postwarning years (2004–2006). The researchers obtained data from a managed health care database which records all claims for prescription treatment and mental health services for its members.

Valluri et al. found that children and adolescents were significantly less likely to use antidepressants after the FDA warning; when comparing treatment within the first 6 months of a new onset depression diagnosis before and after the warning, they observed a 7% decrease in antidepressant use after the warning (42% prewarning and 35% postwarning) (78). However, this was only true for youth with less severe depression diagnoses. Consistent with Kafali et al.’s (75) findings, Valluri et al. found that children and adolescents with major depressive disorder did not experience a significant reduction in the likelihood of receiving antidepressants after the suicidality warning.

While treatment with antidepressants decreased following the warning, Valluri et al. found that psychotherapy increased (from 64 to 67%). There was also significant increase in psychotherapy without concomitant antidepressant use: the percentage of youth who were treated with psychotherapy alone increased from 37 to 44%. Additionally, they observed a trend of increasing psychotherapy treatment with or without concomitant antidepressant use from late 2004 through 2006. The likelihood of a psychotherapy visit after the warning significantly increased for children by 31% and for adolescents by 19%.

Valluri et al.’s findings (78) indicate that the FDA suicidality warning had beneficial effects for prescribing practices in the treatment of youth with depression, consistent with Kafali’s findings (75). It appears that the suicidality warning did what it was intended to do: it encouraged medical service providers to exercise caution when prescribing antidepressants to youth, being more alert to serious medication-linked risks. Not only did medical providers use more care and restraint when prescribing antidepressants, which caused a decline in antidepressant use after the warning, but providers were more inclined to consider prescribing psychotherapy as an effective alternative to antidepressant treatment. Indeed, after the warning, there was a significant increase in the likelihood that youth with new-onset depression diagnoses would be treated with psychotherapy. It is apparent from Kafali et al.’s findings that the effects of the warning did not persist, and that within 5 years of the warning, antidepressant use returned to the prewarning levels (75).

We note briefly that Libby et al. found somewhat differing results regarding trends in treatment after the Black Box warnings (79). They compared post-warning antidepressant prescription rates with expected prescribing rates based on trends over the years leading up to the warning. They observed a 10% reduction in antidepressant prescriptions for depressed youth by June 2007. However, they did not break down antidepressant prescriptions by type of depression diagnosis. Later research found that antidepressant prescribing trends among American youth have increased from 2006 to 2012 (66).

Ecological studies possess little ability to demonstrate cause-effect relationships. As suicide is relatively rare, suicide rates are prone to fluctuation. Thus, researchers can select various time points and then cherry-pick data from various datasets that could support a conclusion that antidepressant prescriptions tend to predict higher or lower rates of suicide among youth. More careful analyses have suggested that clinicians responded to regulatory warnings by writing fewer prescriptions for less impaired (and thus less at risk for suicide) youth while not changing their rate of antidepressant prescriptions among more severely impaired children and adolescents. Regardless of short-term outcomes after regulatory warnings, rates of antidepressant prescriptions for youth have increased in recent years in both the USA and Europe (66).

#### Recent Trends in Adolescent Suicidal Behavior

While fully recognizing the limitations of ecological studies examining the relationship between real-world suicidality and rates of antidepressant prescriptions, we present a few observations based on more recent trends in the United States.

Over 176,000 adolescents participated in the National Surveys on Drug Use and Health (NSDUH) between 2005 and 2014. Mojtabai et al. examined the prevalence of depression among adolescents and what treatments were received during this period (80). In 2005, 16.5% of depressed adolescents reported taking medication for depression, which increased to 20.1% by 2014. Additionally, the prevalence of depression among adolescents increased from 8.7 to 11.3% over the course of the study. Thus, more adolescents reported being depressed and a higher percentage of depressed adolescents reported taking medication for depression.

Plöderl and Hengartner examined the same NSDUH data set to examine possible relationships between antidepressant prescription rate and the rate of self-reported suicide attempts. They compared the relationship between rates of antidepressant prescribing and suicide for depressed youth and for non-depressed youth. This makes good sense given than antidepressants are often prescribed for anxiety disorders and other conditions in addition to being prescribed for major depression. They found a significant upward change in antidepressant prescription rate starting in 2012, then a significant change upward for suicide attempts beginning in 2013. Between 2004 and 2016, they found a) a strong positive correlation (*r* = .76) between suicide attempt rates and antidepressant prescription rates among nondepressed youth and b) a moderate positive correlation (*r* = .41) between suicide attempt rates and antidepressant prescription rates among depressed youth (81). Relatedly, a strong trend toward elevated suicide attempts has been observed in patient encounters at American children’s hospitals between 2008 and 2015, where the percentage of children’s encounters which were due to either suicidal ideation or suicide attempt went from 0.66% in 2008 to 1.82% in 2015 (82).

Increased prevalence of depression in youth may be the most relevant variable to explain the link between increasing antidepressant prescriptions and increased suicide attempts in depressed adolescents. However, this would not explain the strong correlation observed between antidepressant prescriptions and suicide among non-depressed individuals. Indeed, while the debate about antidepressant warnings tends to focus narrowly on depressed youth, it is worth remembering that antidepressants are linked to greater suicidality in clinical trials among anxious, as well as depressed youth (9).

We recognize that the observed relationship between a) increasing antidepressant treatment of adolescent depression and b) increasing self-reported adolescent suicide attempts and increasing rates of hospital visits for suicidal ideation/attempts could be due to extraneous variables. As we have noted throughout this paper, ecological studies have quite limited ability to draw cause-effect conclusions. However, if one takes ecological studies seriously when they purportedly demonstrate that fewer antidepressant prescriptions relate to greater suicidality or completed suicide (14, 15, 29, 83), then one should also take such studies seriously when they suggest antidepressant-linked suicidality risk (81). Due to their methodological limitations, we consider ecological studies to provide generally weak (and potentially misleading) evidence. But it is clear that the initial claims of regulatory warnings leading to decreased antidepressant prescriptions leading to a cascade of youth suicides were incorrect. Analysis of more reliable longer-term trends shows that increased antidepressant prescriptions have coincided with more suicide attempts among American adolescents.

### Case-Control Studies

Olfson, Marcus, and Shaffer examined the relationship between antidepressant use and suicidality *via* a case control study which examined suicide risk in patients treated with antidepressants versus those not treated with antidepressants (84). The sample consisted of children and adolescents with severe depression who had received inpatient treatment for depression, either with or without antidepressant treatment, based upon Medicaid data from January 1999 to December 2001. Cases of suicide attempts and completed suicides were matched to controls who had not attempted or completed suicide by age, sex, race, and presence or absence of recent suicide attempt or substance abuse disorder. The sample included 263 youth with suicide attempts and 1,241 matched controls who did not attempt suicide, and eight cases of completed suicide among youth matched with 39 controls who did not commit suicide.

One could sensibly argue that antidepressants are more likely to be prescribed to youth with severe depression, and youth with severe depression are also at higher risk of suicide; following this logic, studies that find an association between antidepressant use and suicide may not convincingly demonstrate that antidepressants increase suicide risk. Such studies may only show that severe depression increases the likelihood of both being prescribed antidepressants and demonstrating suicidality. Olfson et al. addressed this potential limitation in two ways: 1) only including participants who had severe depression—all participants had been hospitalized for their depression and had a “high and comparable level of illness severity” (p. 866) and 2) matching cases to controls on a variety of suicide risk factors, including days since hospital discharge and presence or absence of recent suicide attempts.

Olfson et al. found that among severely depressed children and adolescents, antidepressants were related to increased risk of completed suicide (odds ratio = 15.62, 95% CI: 1.65 to infinity) and attempted suicide (Odds ratio = 1.52, 95% CI: 1.12–2.07). While Olfson found a significant association between suicide and antidepressant for youth, the association was not significant for adults using antidepressants. Olfson et al. (84) findings support the FDA warning on the increased risk of suicidality for youth using antidepressants. There are two key limitations: a) the use of only severely depressed participants and b) the very small number of completed suicides. Thus, the results should be interpreted cautiously. With these limitations in mind, the results align with regulatory suicidality warnings.

A systematic review of observational cohort and case-control studies found that adolescents who take SSRIs are at a statistically significantly greater risk of completed suicide and suicide attempts relative to matched controls who do not take SSRIs (85). Such studies certainly run the risk of confounding by indication, such that more severely disturbed people may be more likely to receive an antidepressant prescription. Studies included in this review made efforts to control for such problems, but it is impossible to know whether such efforts were entirely successful. Nonetheless, we find it interesting that these results point in the direction of increased antidepressant-related suicidality risk.

### Limitations

Our paper is tempered by some limitations. We did not perform a systematic literature search, so it is certainly possible that we missed some important, relevant studies on the relationship between antidepressants and suicidality among youth. That being said, we examined important problems in several pertinent studies, some of which have been cited widely in the literature. At this point, a systematic review of anything other than the aforementioned clinical trials would be impossible given the different methodologies, participants, and measures used across studies. The lack of standardized suicidality measures certainly complicates the study of antidepressant-linked suicidality.

While the C-SSRS measure of treatment-emergent suicidality has become incorporated into recent antidepressant clinical trials, use of this measure does not solve the problem of inaccessible underlying clinical trial data which are sometimes reported inaccurately in medical journals (51, 54, 57). In addition, while there is some supportive evidence for the C-SSRS’s validity (42, 43), some authors have raised concerns about its wording and validity (45). A recent paper from Silverman and De Leo, two leaders in the field of suicidology, stated: “No classification system has been fully tested to date in terms of determining whether the terms (and their definitions) actually describe in an accurate way the phenomenology and, in fact, distinctly classify the range of thoughts, actions, and behaviors associated with the suicidal process” [(86) pg. 83]. Further work is clearly needed in developing a valid coding scheme for suicidality.

### Managing Antidepressant-Induced Suicidality

The term “activation” refers to a group of adverse events which include mania, irritability, insomnia, agitation, restlessness, or excessive excitability (87, 88). These events occur at much higher rates for youth taking antidepressants relative to placebo (88). There is some support for a link between treatment-emergent activation and suicidality (89), though either suicidality or activation can emerge independently, and activation seems more common than suicidality (87, 90).

Regardless of whether activation is present or not, there is little empirical guidance on how clinicians should proceed in cases of treatment-emergent suicidality. Rather than automatically assuming that suicidality is caused by depression or another mental health problem, a clinician who knows that antidepressants can cause suicidality is in the position to carefully consider whether treatment-emergent suicidality could be an iatrogenic reaction.

Pompili et al. reported on 10 adult patients who became suicidal while taking an antidepressant and whose suicidality was then resolved after changing the course of treatment (91). In each case, the offending antidepressant was either discontinued or the dosage was lowered. Additional medications were utilized to treat the newly emergent suicidality, including benzodiazepines, lithium, anticonvulsants, and/or antipsychotics. Dosage reduction or drug discontinuation seems like obvious first steps to managing drug-induced suicidality, with the addition of other interventions as appropriate to manage suicidality and any other related mental health problems. Future research on the management of iatrogenic suicidality is sorely needed.

## Conclusion

RCTs are considered the most powerful avenue to demonstrate cause-effect relationships. In the case of antidepressants for youth, RCTs have demonstrated increased risk for suicidal thoughts and behaviors (9). Based on this evidence, various regulatory agencies worldwide issued warnings. Using data from rather poorly designed methodology or cherry-picking data from a time series association between antidepressant prescriptions and suicide rates among youth, some have argued that regulatory warnings caused a decrease in antidepressant prescriptions, which then caused more suicides among youth (14, 15, 29). However, a more careful analysis yields several observations:

- The most powerful evidence of efficacy for antidepressants over placebo in depressed youth is on clinician-rated depression measures, where their benefit is d = .20, literally the smallest of small effects according to Cohen’s convention (92).
- On self-reports of depressive symptoms, or across measures of quality of life, global mental health, or autonomy, antidepressants have failed to beat placebo (77).
- The drug industry has under-reported antidepressant-related harms, including of suicidality (54, 55, 57).
- Blind review of adverse event reports commissioned by the FDA found increased likelihood of suicidal ideation/behavior on antidepressants relative to placebo (9). Gibbons et al. found that fluoxetine does not increase suicidality on the CDRS-R suicidality rating item relative to placebo among depressed youth (17). However, the CDRS-R does not seem well-suited to detect suicidal events (20).
- Studies done after the FDA review that have systematically assessed suicidality are few in number, limiting the conclusions that can be drawn. In one such trial of escitalopram, two suicidal events were inappropriately attributed to placebo and placebo fared better than escitalopram in reducing suicidal ideation on a self-report suicidality measure (26, 46). Other more recent studies have not reported notable differences in treatment-emergent suicidality between antidepressants and placebo.
- Ecological time-series studies are only weakly equipped to examine drug-induced suicidality, particularly when the underlying risk is small at a population level and trends are not observed over a long time period. Data can be cherry-picked from such studies, primarily by choice of time points, to support any claim one wants to bolster.
- Some ecological and time-series studies have made claims that fall well beyond their results, and these studies are typically done over too short of a period to account for any longer-term trends (14, 15, 29).
- A more recent time-series study across 2004–2016 found a notable, statistically significant relationship between rising antidepressant prescription rates and increasing suicide attempts among American adolescents (81).
- Case-control and observational cohort studies have found increased risk of suicide attempts and completed suicides among youth taking SSRIs relative to youth not receiving such treatment (85).

Based on the sum of this evidence, regulatory warnings regarding antidepressant-linked suicidality are clearly warranted. When a clear body of evidence points to increased treatment-linked risk, patients and healthcare providers should be made aware of these risks. To suggest otherwise both breaches the ancient injunction of *primum non nocere* (first, do no harm) and is not aligned with the practice of evidence-based medicine.

## Author Contributions

GS, TS-S, and PP conceived and designed the study. GS, TS-S, and PP gathered relevant data. GS wrote the first draft of the manuscript. TS-S and PP wrote sections of the manuscript, contributed to manuscript revision and have read and approved the submitted version.

## Conflict of Interest

GS has holdings in Vanguard Healthcare, a mutual fund that invests heavily in pharmaceutical firms.

The remaining authors declare that the research was conducted in the absence of any commercial or financial relationships that could be construed as a potential conflict of interest.
